# Cystatin C Is Not Causally Related to Coronary Artery Disease

**DOI:** 10.1371/journal.pone.0129269

**Published:** 2015-06-09

**Authors:** Patrik Svensson-Färbom, Peter Almgren, Bo Hedblad, Gunnar Engström, Margaretha Persson, Anders Christensson, Olle Melander

**Affiliations:** 1 Trelleborg Hospital, Department of Internal Medicine, Trelleborg, Sweden; 2 Institution of Clinical Sciences, Lund University, Malmö, Sweden; 3 Department of Internal Medicine, Skåne University Hospital, Malmö, Sweden; 4 Department of Nephrology, Skåne University Hospital, Malmö, Sweden; Innsbruck Medical University, AUSTRIA

## Abstract

**Background:**

Strong and independent associations between plasma concentration of cystatin C and risk of cardiovascular disease (CVD) suggests causal involvement of cystatin C.

**Aim:**

The aim of our study was to assess whether there is a causal relationship between plasma concentration of cystatin C and risk of coronary artery disease (CAD) using a Mendelian Randomization approach.

**Methods:**

We estimated the strength of association of plasma cystatin C on CAD risk and the strength of association of the strongest GWAS derived cystatin C SNP (rs13038305) on plasma cystatin C in the population-based Malmö Diet and Cancer Study (MDC) and thereafter the association between rs13038305 and CAD in the MDC (3200 cases of CAD and 24418 controls) and CARDIOGRAM (22233 cases of CAD and 64762 controls).

**Results:**

Each standard deviation (SD) increment of plasma cystatin C was associated with increased risk of CAD (OR = 1.20, 95% CI 1.07–1.34) after full adjustment. Each copy of the major allele of rs13038305 was associated with 0.34 SD higher plasma concentration of cystatin C (P<1 x 10^-35^), resulting in a power of >98% to detect a significant relationship between rs13038305 and CAD in MDC and CARDIOGRAM pooled. The odds ratio for CAD (per copy of the major rs13038305 allele) was 1.00 (0.94–1.07); P = 0.92 in MDC, 0.99 (0.96–1.03); P = 0.84 in CARDIOGRAM and 1.00 (0.97–1.03); P = 0.83 in MDC and CARDIOGRAM pooled.

**Conclusion:**

Genetic elevation of plasma cystatin C is not related to altered risk of CAD, suggesting that there is no causal relationship between plasma cystatin C and CAD. Rather, the association between cystatin C and CAD appears to be due to the association of eGFR and CAD.

## Introduction

Human γ-trace, also called post-γ-globulin, was first described in 1961 and the name Cystatin C was proposed in 1984[1]. Cystatin C is an active cysteine protease inhibitor and is the most abundant cysteine protease inhibitor of the cystatin C superfamily and is found in all body fluids and is expressed in most of the nucleated cells within the human body and is proposed to be a better marker of renal function than creatinine[2–4]. Cystatin C has been suggested to be a protective protein and prevents brake down of the extracellular matrix by preventing enzymatic cleavage of connective tissues by cathepsins[5–7].

Cystatin C, a non-glycosylated cationic low molecular weight protein (Mw 13 343 Da) is an inhibitor of cysteine proteinases. This protein is eliminated by glomerular filtration, reabsorbed and catabolized in proximal renal tubular cells without tubular secretion[8, 9] and thus has the characteristics of an ideal endogenous GFR marker[10]. Compared to creatinine, cystatin C is less affected by age, sex and muscle mass, which may explain why cystatin C seems to better estimate true renal function than plasma levels of creatinine or a creatinine based estimated glomerular filtration rate (eGFR)[11, 12]. However, cystatin C is at some extent affected by non-renal factors such as age, sex, diabetes, high C-ractive protein, high white cell blood count and low serum albumin levels which might affect estimated GFR when using cystatin C, which in some cases should be taken into account when calculating eGFR[13].

Increased plasma level of cystatin C is associated with increased risk of cardiovascular disease (CVD) and mortality in the elderly [14–16] and in different patient populations [17–22]. The general theory of why elevated cystatin C is associated with increased cardiovascular risk is that it represents impaired renal function, which in turn contributes to increased risk of cardiovascular disease. However, in several studies cystatin C has been associated with CVD even within normal ranges of eGFR [23, 24], suggesting existence of GFR independent cystatin C mediated CVD risk. For example, we recently reported that elevated cystatin C promotes progression towards a dysmetabolic state[25]. Intriguingly, an anti-atherogenic role of cystatin C has also been suggested as cystatin C inhibits pro-inflammatory cathepsins in the vasculature with one hypothesis being that the high cystatin C which precedes CVD represents a compensatory rise to counterbalance the effect of cathepsins [6]. Shlipak et al also speculated over the toxic effects of cystatin C as a cause of CVD and not only the relation to GFR[15].

Genome-wide Association studies (GWAS) for genetic determinants of cystatin C have pinpointed the strongest signal of the genome associating with variation of plasma concentration of cystatin C at the cystatin C locus on chromosome 20, represented by the single nucleotide polymorphism (SNP) rs13038305. Of note, this SNP was not associated with creatinine-based measures of renal function and thus likely to affect cystatin C plasma concentration independently of renal function [26].

We hypothesized that cystatin C may exert GFR independent effects increasing the risk of coronary artery disease (CAD) and thus set out to test whether or not there is a causal relationship between cystatin C and risk of CAD using a Mendelian Randomization approach where the strongest genetic signal for plasma concentration of cystatin C identified thus far (rs13038305) was related to risk of CAD.

## Materials and Methods

### The Malmö Diet and Cancer Study: Subjects, phenotyping, genotyping and CAD events

#### MDC and MDC-CC

The Malmö Diet and Cancer study (MDC) is a population-based prospective cohort consisting of 30 447 subjects (DNA available on n = 28 767) surveyed at a baseline examination in 1991–1996 [27]. From this cohort, 6 103 subjects were randomly selected to be studied for the epidemiology of carotid artery disease of whom 5400 subjects came in the fasted state. This subsample of the MDC is referred to as the MDC cardiovascular cohort (MDC-CC) and was examined 1991–1994 [28].

#### Genetic analyses

DNA was extracted from frozen granulocytes or buffy coats with the use of QIAamp-96 spin blood kits (QIAGEN, Stockholm, Sweden) at the DNA extraction facility supported by SWEGENE. We successfully genotyped the plasma cystatin C associated SNP rs13038305 at the cystatin C locus on chromosome 20 in 27 618 subjects of the MDC. Primers and probes were custom synthesized by Applied Biosystems (Foster City, CA) according to standard recommendations for the AB Prism 7900HT analysis system, and genotyped with polymerase chain reaction-based TaqMan method[29].

#### Assays and phenotyping

Within the subsample of MDC-CC we analysed plasma concentration of cystatin C and creatinine in fasting plasma samples in 4743 subjects who also completed genotyping of the rs13038305. Levels of plasma cystatin C were measured using a particle-enhanced immunonephelometric assay (N Latex Cystatin; Dade Behring, Deerfield, Illinois) and presented in mg/L[15]. Plasma creatinine is presented in μmol/L, and was analyzed with the Jaffé method and was traceable to the International Standardisation with Isotope Dilution Mass-Spectometry (IDMS). eGFR was assessed with the formulae of Cockroft-Gault creatinine clearance (mL/min/1.73 m^2^) = (140-age) x weight in kg x 1.23 / P-creatinine (x 0.85 if female) (CG)[30]. CG was adjusted for body surface area (BSA) to CG/BSA in mL/min/1.73 m^2^ by estimating BSA for all persons from the duBois formulae (BSA in m^2^ = 0.00718 x (length in cm)^0,725^ x (weight in kg)^0,425^). MDRD was calculated from the four-variable MDRD GFR formulae (mL/min/1.73 m^2^) = 175 x (P-creatinine / 88.4) ^−1.154^ x age ^−0.203^ (x 0.742 if female)[31]. Adjustment for race was not applicable in this homogenous cohort of Caucasian participants.

All MDC participants underwent a medical history and physical examination. Blood pressure was measured using a mercury-column sphygmomanometer after 10 minutes of rest in the supine position. Hypertension was defined as systolic or diastolic blood pressure ≥140/90 mmHg or use of antihypertensive medication. Diabetes mellitus was defined as a fasting whole blood glucose >109 mg/dl (6.0 mmol/L), a self-reported physician diagnosis of diabetes, use of anti-diabetic medication or having been diagnosed in local or national Swedish diabetes registries, as described previously [32]. Cigarette smoking was elicited by a self-administered questionnaire, with current cigarette smoking defined as any use within the past year.

Within the MDC-CC we measured fasting total cholesterol, HDL cholesterol, and triglycerides according to standard procedures at the Department of Clinical Chemistry, University Hospital Malmö. LDL cholesterol was calculated according to Friedewald’s formula.

#### Assessment of CAD events

Retrieval of prevalent (occurring before the baseline exam of the MDC) and incident (occurring after the baseline exam of the MDC) cases of CAD were identified through linkage of the 10-digit personal identification number of each Swedish citizen with three registers: the Swedish Hospital Discharge Register, Swedish Coronary Angiography and Angioplasty Registry (SCAAR) and the Swedish Cause of Death Register. The registers have been previously described and validated for classification of outcomes [33, 34]. Follow-up for retrieval of CAD cases extended to June 30, 2009.

CAD was defined as fatal or non-fatal myocardial infarction (MI), death from ischemic heart disease, CABG or PCI, whichever came first. MI was defined on the basis of International Classification of Diseases 9th and 10th Revisions (ICD9 and ICD10) codes 410 and I21, respectively. Death due to ischemic heart disease was defined on the basis of codes 412 and 414 (ICD9) or I22–I23 and I25 (ICD10). CABG was identified from national Swedish classification systems of surgical procedures, the KKÅ system from 1963 until 1989 and the Op6 system since then. CABG was defined as a procedure code of 3065, 3066, 3068, 3080, 3092, 3105, 3127, 3158 (Op6) or FN (KKÅ97). PCI was defined based on the operation codes FNG05 and FNG02.

All MDC participants gave informed consent and the study was approved by the local ethics committee, Lund University, Sweden (LU-51-90).

### CARDIoGRAM

The CARDIoGRAM study is a meta-analysis of 14 GWAS studies of CAD comprising 22 233 individuals with CAD and 64 762 controls of European descent [35]. Included cohorts and methodology has been described in details previously [35].

Of note, 185 participants (86 CAD cases and 99 control subjects) of the MDC were also participants in the Myocardial Infarction Genetics Consortium [36], later included in the CARDIOGRAM study. These 185 subjects remained included in the CARDIOGRAM analyses but were excluded in all MDC analyses of the current study.

### Statistics and power

We first estimated the strength of association of the association between plasma cystatin C (expressed as per 1 SD increase) and risk of incident CAD, after exclusion of prevalent CAD cases, using logistic regression in the MDC-CC. Cystatin C was transformed with the natural logarithm before analysis due to skewness to the right of its distribution. Two models adjusting for age and sex (model 1) and full risk factor adjustment (age, sex, hypertension, smoking, diabetes, LDL- and HDL-cholesterol (model 2), were applied. Thereafter, again in the MDC-CC, we used linear regression to test the association between the rs13038305 (additive models with the major allele, which previously has been shown to be associated with higher plasma cystatin C, coded C) and standardized values of LN-transformed cystatin C (dependent variable) adjusting both according to model 1 and model 2 (see above). Based on the plasma cystatin C vs. CAD and the rs13038305 vs. plasma cystatin C relationships we then calculated the power to detect association between rs13038305 and CAD in MDC + CARDIoGRAM, assuming that the rs13038305 genetic effect on plasma cystatin C would be independent of renal function and that the relationship of the rs13038305 genetic plasma cystatin C elevation on CAD would be proportional to the over-all relationship between plasma cystatin C and CAD.

Finally, we performed logistic regressions testing the association between rs13038305 and CAD (including both prevalent and incident cases of CAD) in a model adjusted for age and sex, in the entire MDC (3200 CAD cases and 24418 control subjects) and in CARDIoGRAM (22 233 CAD cases and 64 762 controls subjects) and meta-analysed the results of the two studies.

SPSS statistical software (version 21.0; SPSS Inc, Chicago, IL, USA) was used for all calculations except for the power calculations which were performed using the PS Power and Sample Size Calculations software version 3.0 (Department of Biostatistics, Vanderbilt University, TN, USA) and the meta-analysis was performed by Inverse-variance-weighted fixed effects model using STATA 11 (STATACorp LP, College Station, TX, USA).

## Results

The clinical characteristics of the MDC and in the subsample of MDC-CC (in whom plasma cystatin C was available) are shown in Table 1. The characteristics of the whole MDC and the MDC-CC were similar.

**Table 1:** Clinical characteristics given for all subjects and for the proportion that also had cystatin C analysed.

	MDC study cohort n = 27618	Part of MDC with analysed cystatin C (MDC-CC) n = 4743
Age, years at inclusion*	58.1 (± 7.7)	57.5 (± 5.9)
Female sex (%)	60.3	59.1
Body Mass Index in kg/m^2^ *	25.8 (± 4.0)	25.7 (± 4.0)
Systolic blood pressure in mmHg *	141.2 (± 20.1)	141.3 (± 18.9)
Diastolic blood pressure in mm Hg *	85.6 (± 10.0)	87.0 (± 9.4)
Hypertension (%)	6.6	7.3
P-Low density lipoprotein in mmol/L *	Not applicable	4. 2 (± 1.0)
P-High density lipoprotein in mmol/L *	Not applicable	1.4 (± 0.4)
Cystatin C in mg/L ^†^	Not applicable	0.76 (0.69–0.85)
CG in ml/min/1.73 m^2^ ^ǂ^ ^†^	Not applicable	75.7 (66.5–86.0)
MDRD in ml/min/1.73 m^2^ ^§^ ^†^	Not applicable	73.7 (65.4–83.1)
Current smoker (%)	26.3	25.4
Diabetes (%)	4.3	4.2

* = Presented as mean ± 1 standard deviation.

^†^ = Presented as median and interquartile range.

^ǂ^ = Estimated glomerular filtration rate with the Cockroft-Gault formula

^§^ = Estimated glomerular filtration rate with the Modification of Diet in Renal Disease (MDRD) formula

### Plasma cystatin C and CAD in MDC-CC

Among MDC-CC subjects free from CAD at the baseline exam (n = 92 excluded due to prevalent CAD), 414 subjects developed CAD during follow-up. Each standard deviation (SD) increment of plasma cystatin C was significantly related to increased risk of CAD during follow-up (Odds Ratio, 95% confidence interval) 1.28, 1.16–1.42 (P = 1.8x10^-6^) after age and sex adjustment and 1.20, 1.07–1.34 (P = 0.001) after full risk factor adjustment according to model 2.

#### rs13038305 in relation to plasma cystatin C and creatinine based measures of GFR

In the MDC-CC, each copy of the major allele of rs13038305 was associated with a highly significant (P<1 x 10^−35^) approximately one third of a SD increase of plasma concentration of cystatin C both in model 1 and model 2. On the contrary, there was no significant association between rs13038305 and creatinine based estimation of renal function (CG and MDRD) (Table 2).

**Table 2:** The cystatin C locus rs13038305 SNP in relation to plasma concentration of cystatin C and eGFR (Cockroft-Gault and MDRD) in the MDC-CC cohort.

	TT (n = 223)	CT (n = 1529)	CC (n = 2991)	B coefficient * (95%CI)	P*	B coefficient^†^ (95%CI)	P^†^
Cystatin C mg/L (median and IQR)	0.69 (0.63–0.78)	0.74 0.67–0.82	0.78 (0.70–0.87)	0.33 (0.29–0.38)	<1x10^-35^	0.34 (0.29–0.38)	<1x10^-35^
CG ml/min/1.73m^2^ (median and IQR) ^ǂ^	75.2 (66.8–87.7)	75.5 (67.1–85.4)	75.9 (66.2–86.0)	-0.02 (-0.07–0.02)	0.33	-0.03 (-0.07–0.01)	0.19
MDRDml/min/1.73m^2^ (median and IQR) ^§^	74.5 (65.6–84.7)	73.6 (65.9–82.6)	73.7 (65.0–83.3)	-0.03 (-0.07–0.02)	0.21	-0.04 (-0.08–0.01)	0.10

CG, creatinine based eGFR according to the Cockroft-Gault formula; MDRD, creatinine based eGFR according to the modification of diet in renal disease formula. Linear regression analyses, the beta-coefficient denotes the difference of standardized values of LN-transformed cystatin C, CG and MDRD per each copy of the coded (major) allele of rs13038305 in additive models.

* = Adjusted for age and sex.

^†^ = Adjusted for cardiovascular risk factors (age, sex, hypertension, diabetes, ldl cholesterol, hdl cholesterol and smoking)

^ǂ^ = Estimated glomerular filtration rate with the Cockroft-Gault formula

^§^ = Estimated glomerular filtration rate with the Modification of Diet in Renal Disease (MDRD) formula

### rs13038305 and CAD

**Fig 1:**
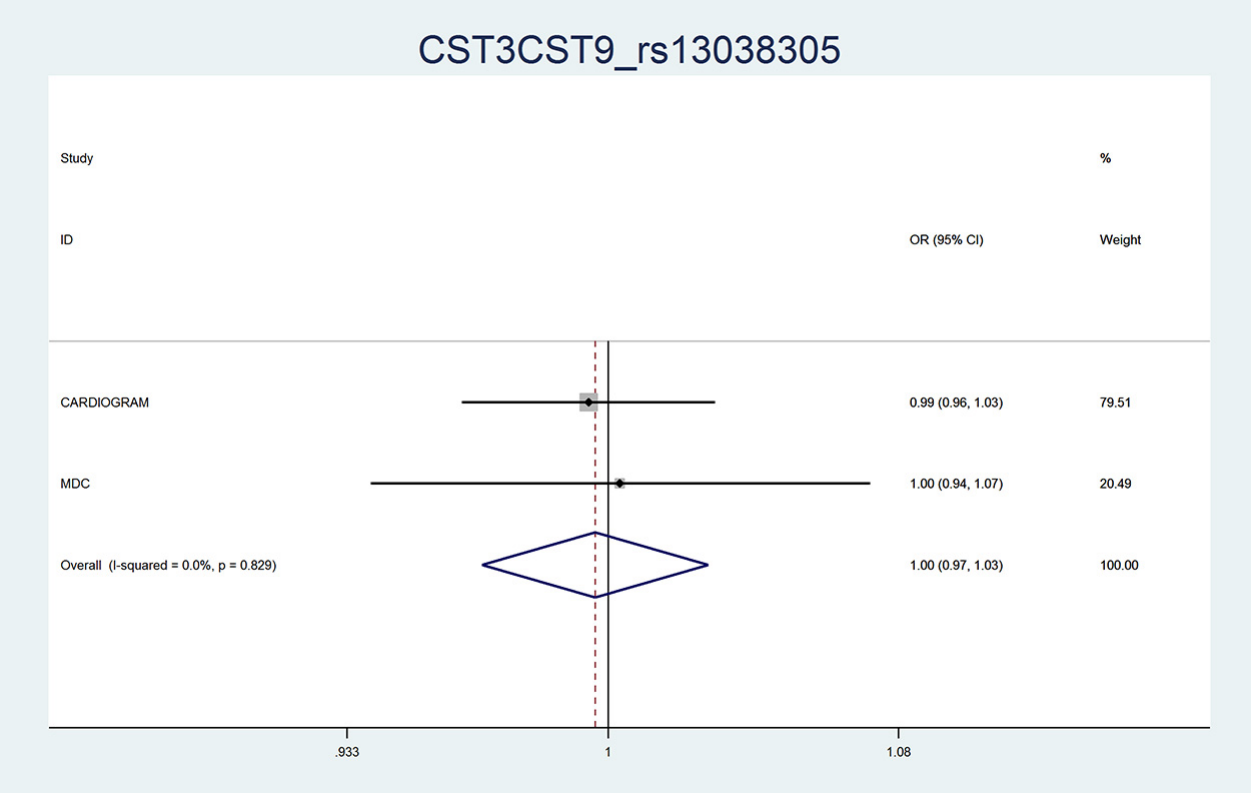
Forest Plot. Meta-analysis of rs13038305 of the cystain C gene and risk of myocardial infarction in the Malmö Diet and Cancer Study (MDC) and CARDIoGRAM. OR = Odds Ratio, CI = confidence interval, weight denotes study weight in the meta-analysis.

At an α = 0.05 and based on the plasma cystatin C vs CAD and rs13038305 vs plasma cystatin C relationships we had 100% power to detect an association between rs13038305 and CAD in the pooled MDC + CARDIoGRAM analysis if we applied the model 1 (age and sex adjusted) effect estimates from the plasma cystatin C vs CAD (OR = 1.28 for CAD per 1 SD increase of cystatin C) whereas the corresponding power estimate was 98% if we applied the model 2 (age, sex, hypertension, smoking, diabetes, HDL and LDL-cholesterol adjustment) effect estimates from the plasma cystatin C vs CAD (OR = 1.20 for CAD per 1 SD increase of cystatin C). However, among the total of 25 433 CAD cases and 89 081 control subjects, the rs13038305 had an odds ratio of 1.00 (95% confidence interval 0.97–1.03) in the pooled analysis and similar strength of association in the two studies analysed separately (Table 3 and Fig 1).

**Table 3:** Risk of coronary artery disease (CAD) according to rs13038305 in MDC, CARDIoGRAM and the pooled data as a meta-analysis.

CST3_CST9 (rs13038305)		
	Odds ratio (95% CI) *	P *
MDC	1.00 (0.94–1.07	0.92
CARDIoGRAM	0.99 (0.96–1.03)	0.84
Meta-analysis	1.00 (0.97–1.03)	0.83

* = Logistic regression analysis, adjusted for age and sex.

## Discussion

We here tested whether there is a causal relationship between cystatin C and risk of CAD using a Mendelian randomization approach by testing whether genetic elevation of cystatin C, measured by a SNP at the cystatin C locus on chromosome 20 robustly associated with plasma cystatin C (rs13038305), alters the risk of CAD in more than 25 000 cases of CAD and almost 90 000 control subjects. Our main finding is that genetic elevation of cystatin C, which in contrast to over-all plasma concentration of cystatin C is not confounded by environmental exposures, is not associated with altered risk of CAD. This strongly implies that cystatin C does not have a causal relationship with risk of CAD. Thus the epidemiological relationship between plasma cystatin C and CAD, despite being statistically independent from CVD risk factors, is likely to be explained by correlation between cystatin C and long-term exposure to CVD risk factors such as hypertension, diabetes and impaired renal function.

When examining whether or not there may be a causal role for cystatin C *per se* in CAD development, selection of the rs13038305 SNP at the cystatin C locus was particularly important mainly for two of reasons. First, as compared with genetic effects on many other previously examined cardiovascular biomarkers, the rs13038305 has a comparatively large effect on its circulating gene product, resulting in approximately one third of a SD increase of plasma cystatin C per copy of the major allele, thus favouring the power of our study. Second, previous studies [26] and our own results in the current study show that rs13038305 does not elevate plasma cystatin C as a result of leading to impaired renal function, neither directly nor through affecting adversely other CVD risk factors which in turn could have caused impaired renal function. The renal function independency of the genetic effect is logical, as the SNP is located at the cystatin C locus and thus can be assumed to affect cystatin C expression and production rather than cystatin C clearance.

It should be emphasized that the function of rs13038305 and the causal relation between rs13038305 and cystatin C are unknown. It is possible that rs13038305 or the causal variant(s) that it represents may influence more than one trait with opposite effects on CAD. This would also result in lack of association between rs13038305 and CAD.

When calculating the power of our Mendelian Randomization study, we based the expected genetic cystatin C elevating effect on CAD on the epidemiological relationship between plasma cystatin C and CAD development. There are several considerations and assumptions linked to such a power calculation. First, if cystatin C itself would mediate detrimental effects on CAD which acted through CVD risk factors, the effect estimate derived from model 1 (age and sex adjusted only) would be most appropriate which would mean that we had close to 100% power to find an association between rs13038305 and CAD. In contrast, if the genetic effect were completely unrelated to CVD risk factors, it would have been more appropriate to apply the effect estimate obtained from model 2 (fully adjusted for CVD risk factors) which resulted in a power of 98% to link genetic effects of cystatin C to CAD risk. We took into account both of these two scenarios and we believe that even the lower power estimate of 98% is sufficient to conclude with relatively large certainty, although not with one hundred percent, that our results exclude a causal relationship between cystatin C and CAD risk.

Although all epidemiological studies point at direct relationship between elevated cystatin C and risk of CVD, there is mechanistic evidence that cystatin C could in fact be protective by inhibiting cathepsins and thus theoretically to reduce matrix degradation and vascular structure and function [6]. This has led to the hypothesis that high cystatin C before CVD events may result from a compensatory increase in cystatin C production in attempts to inhibit an ongoing disease process. In fact, there are previous examples from cardiovascular biomarkers, which turned out to be protective but are elevated before CVD presents due to such compensatory increase of secretion. One of those examples relates to natriuretic peptides which consistently are high in individuals at high risk, however, Mendelian randomization studies revealed that genetic atrial natriuretic peptide deficiency instead seems to be a risk factor [37]. Still, if cystatin C instead would be causally protective against CAD, our study would likely have unmasked that relationship as well as we had 80% power to detect an odds ratio for CAD of 0.96 in the analyses associating rs13038305 with CAD.

## Conclusion

Genetic elevation of plasma cystatin C is not related to altered risk of CAD, suggesting that there is no causal relationship between plasma cystatin C and altered risk of CAD. Our results suggest that high level of cystatin C before CAD occurs as result of co-variation with CVD risk factors and impaired renal function. The lack of a causal relationship does not encourage development of interventions targeted at the cystatin C system in attempts to prevent CAD.
